# The effect of Benzothiazolone‐2 on the expression of Metallothionein‐3 in modulating Alzheimer's disease

**DOI:** 10.1002/brb3.799

**Published:** 2017-08-15

**Authors:** Sudeep Roy, Jaromir Gumulec, Akhil Kumar, Martina Raudenska, Mohd Hassan Baig, Hana Polanska, Jan Balvan, Mansi Gupta, Petr Babula, Jan Odstrčilík, Inho Choi, Ivo Provaznik, Michal Masarik

**Affiliations:** ^1^ Department of Biomedical Engineering Faculty of Electrical Engineering and Communication Brno University of Technology Brno Czech Republic; ^2^ Central European Institute of Technology Brno University of Technology Brno Czech Republic; ^3^ Department of Physiology Faculty of Medicine Masaryk University Brno Czech Republic; ^4^ Biotechnology Division CSIR–Central Institute of Medicinal and Aromatic Plants Lucknow India; ^5^ School of Biotechnology Yeungnam University Gyeongsan Korea; ^6^ Department of Pathological Physiology Faculty of Medicine, Masaryk University Brno Czech Republic

**Keywords:** Alzheimer's disease, flow cytometry, immunodetection, metallothionein‐3, molecular dynamics, qRT‐PCR

## Abstract

**Introduction:**

Metallothioneins (MTs) are a class of ubiquitously occurring low‐molecular‐weight cysteine‐ and metal‐rich proteins containing sulfur‐based metal clusters. MT‐3 exhibits neuro‐inhibitory activity. The possibility to enhance the expression of MT‐3 or protect it from degradation is an attractive therapeutic target, because low levels of MT‐3 were found in brains of Alzheimer's disease (AD) patients.

**Objectives:**

The primary objective of this study was to test an enhancement of MT‐3 cellular concentration after MT‐3 binding treatment, which could prevent MT‐3 degradation.

**Methods:**

MTT assay, flow‐cytometry, fluorescence microscopy, quantitative real‐time polymerase chain reaction, and immunodetection of MT3 were used for analysis of effect of STOCK1N‐26544, STOCK1N‐26929, and STOCK1N‐72593 on immortalized human microglia‐SV40 cell line.

**Results:**

All three tested compounds enhanced concentration of MT‐3 protein in cells and surprisingly also mRNA concentration. IC50 values of tested molecules exceeded about ten times the concentration that was needed for induction of MT‐3 expression. The tested compound Benzothiazolone‐2 enhanced apoptosis and necrosis, but it was not of severe effect. About 80% of cells were still viable. There was no serious ROS‐generation and no severe decrease in mitochondria numbers or stress induced endoplasmic reticulum changes after test treatments. The selected compound showed stable hydrophobic and electrostatic interaction during MT‐3 ligand interaction.

**Conclusion:**

Benzothiazolone‐2 compounds significantly enhanced MT‐3 protein and mRNA levels. The compounds can be looked upon as one of the probable lead compounds for future drug designing experiments in the treatment of Alzheimer's disease.

## 1 INTRODUCTION

Alzheimer's is the most common form of dementia; it is a term for memory damage and other intellectual capacities that are serious enough to impede daily life. Alzheimer's disease accounts for 60 to 80 percent of dementia cases. The neuropathological hallmarks of Alzheimer disease (AD) are comprised of “positive” lesions such as amyloid plaques, cerebral amyloid angiopathy, neurofibrillary kinks, glial responses and “negative” lesions such as neuronal and synaptic loss (Haass & Selkoe, 2007; Iqbal, Liu, Gong, Alonso Adel, & Grundke‐Iqbal, 2009). The neurochemical elements responsible for this age‐linked compulsive advance are still poorly described. The growing evidence supports a significant role for biometals such as copper (Cu), iron (Fe), and zinc (Zn) in Aβ accretion and neuronal relapse (Bush & Tanzi, 2008; Lovell, Robertson, Teesdale, Campbell, & Markesbery, 1998; Miu & Benga, 2006; Roberts, Ryan, Bush, Masters, & Duce, 2012; Zatta, 2008). The plasma Cu/MT ratio was found to be symptomatic of disease advancement in AD patients. This connotation hints at a probable connection between some pathological features of the disease and the MTs expression.

Metallothioneins (MTs) are a family of low‐molecular weight and cysteine‐rich proteins present in all eukaryotes. The MT family is encompassed of four main members (MT‐1 to MT‐4) with multiple isoform subclasses. MT‐1 and MT‐2 are extensively expressed in almost all tissues, MT‐3 mainly in the central nervous system (CNS), whereas MT‐4 is present in squamous epithelial tissue (Palacios, Atrian, & Capdevila, 2011).

Many studies show that MT‐3 mRNA is downregulated in AD brains (Barnham et al., 2004; Carrasco et al., 1999; Kim, Nam, Jeon, Han, & Suk, 2012; Naruse et al., 1994; Streit, 2004; Uchida, Takio, Titani, Ihara, & Tomonaga, 1991; Yu, Lukiw, Bergeron, Niznik, & Fraser, 2001); this might, therefore, contribute to the upsurge of abnormal neuronal development that is associated with the disease. Degradation and deficiency in MT‐3 may lead to severe disruption of lysosomal biogenesis and cytoskeleton dynamics. Dysfunction of lysosomes and actin cytoskeleton in MT‐3‐null astrocytes contributed to accumulation of toxic Aβs proteins and other damaged proteins. Apart from the function of MT‐3 in lysosomes and cytoskeleton, homeostasis of metal ions managed by MT‐3 may also play a key role in AD (Bonda et al., 2011).

Furthermore, MT‐3‐null astrocytes had low level of zinc in lysosome and presented autophagy defect. Autophagy seems to be abnormal due to alteration of the endo‐lysosomal pathway, which impairs fusion of autophagosomes with lysosomes. In addition, a recent report details the contribution of mitophagy, a specialized form of autophagy that removes damaged mitochondria in AD (Moreira et al., 2007).Interestingly, the activation of autophagy can promote degradation of APP/Aβ and reduce tau pathology (Nixon, 2007). Therefore, the autophagy pathway is considerably more complex in AD because it is simultaneously induced and impaired. APP/Aβ can be generated in autophagosomes at low levels in a physiological environment; however, during disease conditions autophagosomes rapidly accumulate in the cell body and axonal terminals due to either impaired autophagic flux or a defected endosomal pathway (Lee, 2009; Lee, Park, Kim, & Koh, 2010).

Martin et al. (2006) showed that in the Tg2576 transgenic mouse model of AD, MT‐3 was less than that found in wild‐type mice. It also establishes the roles of MT‐3 and Nnos in Alzheimer's disease. The pathological changes that develop in this mode might be responsible for the degradation of MT‐3.

Roy, Kumar, Baig, Masarik, and Provaznik (2015) reported that natural‐based compounds were effective in modulating Alzheimer's disease. The *in silico* findings (which include studies of both pharmacodynamics and pharmacokinetic properties) show that MT‐3 was able to bind with STOCK1N‐26544, 26929, and 72593 compounds. The former two compounds contain Benzothiazolone‐2 as a fragment (3‐(2‐oxobenzo[d]thiazol‐3(2H)‐yl)propanoicacid (propanoic acid of Benzothiazolone‐2) was used in the synthesis). It is obtained from *Micrococcus* sp. found in *Tedania ignis*, whereas the latter compound is a modified base of tryptamine obtained widely in plants, such as *Acacia* spp., *Lens esculenta*,* Prosopsis juliflora*, as well as from the fungi *Ponaeolus foenisicii*,* Coprinus micaceus* and from the gorgonian *Paramuricia chamaeleon*.

The current work validates the *in silico* findings for the compounds. Experiments were performed to see the effect of these compounds on live immortalized human microglia cells (SV40) and their effect on MT‐3 cellular concentration. The study also includes a binding free‐energy calculation of the tested natural compound complexes and the standard controls through molecular dynamics simulations.

## 2 MATERIALS AND METHODS

### 2.1 Cell cultures and cultured cell conditions

The immortalized human microglia‐SV40 (Applied Biological Materials Inc., Richmond, BC, Canada) were used in this study. These cells are positive for NGF [nerve growth factor (Beta Polypeptide)], Iba1 ionized calcium‐binding adapter molecule 1, TREM2 (triggering receptor expressed on myeloid cells 2), CD11b, and CD68. The immortalized human microglia was cultured in Prigrow III Medium with 10% fetal bovine serum in collagen‐coated flasks. The medium was supplemented with penicillin (100 U/ml) and the cells were maintained at 37°C in a humidified incubator with 5% CO_2_.

### 2.2 RNA isolation and reverse transcription

TriPure Isolation Reagent (Roche, Basel, Switzerland) was used for RNA isolation. RNA samples without reverse transcription were used as the negative control for qRT‐PCR, in order to exclude DNA contamination. The isolated RNA was used for the cDNA synthesis. RNA (1000 ng) was transcribed using the Transcriptor First Strand cDNA Synthesis Kit (Roche), which was applied according to the manufacturer's instructions. The cDNA (20 μl) prepared from the total RNA was diluted with RNase‐free water to 100 μl; 5 μl was then directly analyzed using the LightCycler^®^480 II System (Roche).

### 2.3 Quantitative real‐time polymerase chain reaction

qRT‐PCR was performed using TaqMan gene expression assays and the LightCycler^®^480 II System (Roche). The amplified DNA was analyzed by the comparative Ct method using β‐actin as a reference gene. The primer and probe sets for ACTB (assay ID: Hs99999903_m1) and MT‐3 (assay ID: Hs01921768_s1) were selected from the TaqMan gene expression assays (Life Technologies, USA). The qRT‐PCR was performed under the following amplification conditions: total volume of 20 μl, initial incubation at 50°C/2 min followed by denaturation at 95°C/10 min; then 45 cycles at 95°C/15 s and at 60°C/1 min. All samples were measured in duplicates.

### 2.4 Immunodetection of MT‐3

Extraction of total protein from the cells was processed using the Pierce^®^ RIPA Buffer (Thermo Scientific, Rockford, IL, USA) in accordance with the protocol provided. All protein concentrations were determined with the Pierce^®^ BCA Protein Assay Kit. Protein samples were diluted to 10 μg/μl, then 1 μl was slowly spotted onto a 0.2μmImmun‐Blot^®^ PVDF membrane (BioRad Laboratories, Hercules, CA, USA) and air‐dried. To block nonspecific binding sites, the membranes were incubated using 5% Blotting‐Grade Blocker (BioRad Laboratories) for one hour at room temperature. They were then incubated using Anti‐MT‐3 antibody (product no. ab76618; Abcam, Cambridge, UK), and with Anti‐β‐actin (product no. ab8227; Abcam). The antibody was diluted 1:1000 using 5% Blotting‐Grade Blocker (BioRad Laboratories).

The membranes were then washed four times in PBS (Sigma‐Aldrich, St. Louis, MO, USA) for five minutes each, before being incubated with a secondary antibody (Peroxidase‐Labeled Anti‐Rabbit IgG (product no. PI‐1000; Vector, Burlingame, CA, USA)). This was diluted 1:2000 using 5% Blotting‐Grade Blocker. After washing in PBS, all blots were visualized on photosensitive film. Densitometric analyses of the membranes were performed using GeneSnap (Syngene, Cambridge, UK).

### 2.5 Cytotoxicity testing – MTT test

The suspension of 5000 cells was added to each well of standard microtiter plates. A volume of 200 μl was transferred to wells 2–11. The medium (200 μl) was added to the first and the last column (1 and 12(control)). The plates were incubated for 2 days at 37°C to ensure cell growth. The medium was removed from columns 2–11. Columns 3–10 were filled with 200 μl of the medium, which contained an increased concentration of tested compound diluted in dimethylsulfoxide (DMSO) (0–1 μmol/l). As a control, columns 2 and 11 were filled with the medium without tested compound.

The plates were incubated for 12 and 24 hr, then the medium was removed and the cells were washed in PBS. Columns 1–11 were filled with 200 μl of medium, which contained 50 μl of MTT (5 mg/ml in PBS) incubated in a humidified atmosphere for four hours at 37°C and then wrapped in aluminum foil. After the incubation, the MTT‐containing medium was replaced with 200 μl of 99.9% DMSO in order to dissolve the MTT‐formazan crystals. Subsequently, 25 μl of glycine buffer was added to all wells; absorbance was immediately determined to be 570 nm (VersaMax microplate reader, Molecular Devices, Sunnyvale, CA, USA). All samples were measured in duplicates.

### 2.6 Confocal microscopy and cell staining

For the purpose of fluorescence microscopy, the cells were cultivated directly on microscope glass slides (75 × 25 mm, thickness 1 mm, MenzelGlässer, Braunschweig, Germany) in Petri dishes in the above‐described cultivation media (see “Cultured cell conditions”). The cells were transferred directly onto the slides, which were then submerged in the cultivation media.

After the treatment, the microscope glass slides with a monolayer of cells were removed from the Petri dishes, rinsed in the cultivation medium without supplementation and PBS buffer and directly used for staining and fluorescence microscopy. The cells were incubated using the following highly specific fluorescent probes:

- Reactive oxygen species were visualized using CellROX Deep Red reagent (Life Technologies, 5 μmol/L, cell‐permeant, life‐cell stain with absorption/emission maxima of 644/665 nm)
- Mitochondria were visualized using MitoTracker Green FM (Life Technologies, 300 nmol/L, cell‐permeant life‐cell stain with absorption/emission maxima of 490/516 nm)
- The endoplasmic reticulum was visualized using ER‐Tracker Red (Life Technologies, 1 μmol/L, cell‐permeant, life‐cell stain with absorption/emission maxima of 587/615 nm).

After incubation (45 min, 37°C, dark), the cells were washed three times with PBS buffer (0.05 mol/L, pH 7.0) and observed under the confocal microscope (Leica TCS SP8 X, Germany) using appropriate excitation and emission wavelengths. Quantitative analysis was performed typically from 10 fields of view. For image analysis, NIS elements BR Analysis (Nikon instruments, Tokyo, Japan) software was used. Average fluorescence intensity was measured in equally confluent fields of view. Results were compared statistically using t‐tests.

### 2.7 Flow cytometry analysis of cell death

Double‐staining with fluorescein isothiocyanate (FITC)/propidium iodide (PI) was undertaken using the Annexin V‐FLUOS‐staining kit (Roche Applied Science) according to the manufacturer's protocols, in order to determine the percentages of viable, apoptotic, and necrotic cells following the exposure to Plumbagin.

Briefly, the cells were harvested by repetitive pipetting and were washed two times with PBS (centrifuged at 2000 rpm for 5 min), resuspended in 100 μl of Annexin‐V‐FLUOS labeling solution and incubated for 15 min in the dark at 15–25°C. Annexin V‐FITC binding was detected by flow cytometry (Partec GmbH, Münster, Germany) (Ex = 488 nm, Em = 533 nm, FL1 filter for Annexin‐V‐FLUOS and FL3 filter for PI).

### 2.8 Thermodynamic studies ‐ MD simulation

The MM‐PBSA (Molecular Mechanics energies combined with Poisson–Boltzmann Surface Area) approach, combined with molecular dynamics (MD) simulations, is widely used in biomolecular complexes as a way to estimate the protein ligand interaction energies and predict binding‐free energies. It also evaluates the relative stabilities of different biomolecular structures. Additionally, it can be used to rescore a set of docked complexes, thereby improving the ability to distinguish between active and inactive lead molecules. The molecular dynamics simulation of MT‐3 docked complexes was performed using the GROMACS 4.5.52 (Berendsen et al., 1995; Lindahl et al., 2001) package, with a standard GROMOS96 force field for 10,000 ps. Binding free‐energy calculations were performed in this work for the ligand‐bound complexes in order to provide further insight into the study.

## 3 RESULTS

### 3.1 The cytotoxic evaluation of benzothiazolone‐2‐containing substances in microglia cells

**Figure 1:**
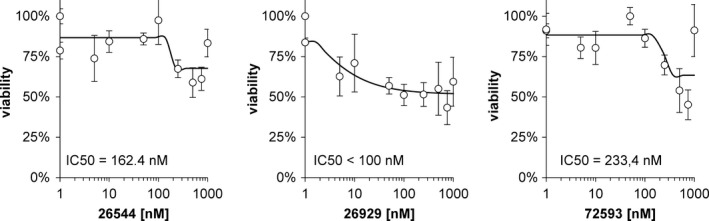
MTT tests for selected compounds on cell lines. The half‐maximal inhibition concentration (IC50) is indicated in the graph. Error bars indicate standard deviations

The IC50 value for STOCK1N‐26544 is 162.4 nmol/L, for STOCK1N‐26929 it is 100 nmol/L and for STOCK1N‐72593 it is 233.4 nmol/L. Thus, STOCK1N‐26929 shows the highest effect on cell metabolic activity. Nevertheless, the IC50 values for all tested molecules exceed the required concentration for the induction of MT‐3 expression by about 10 times (see Figure 1).

**Figure 2:**
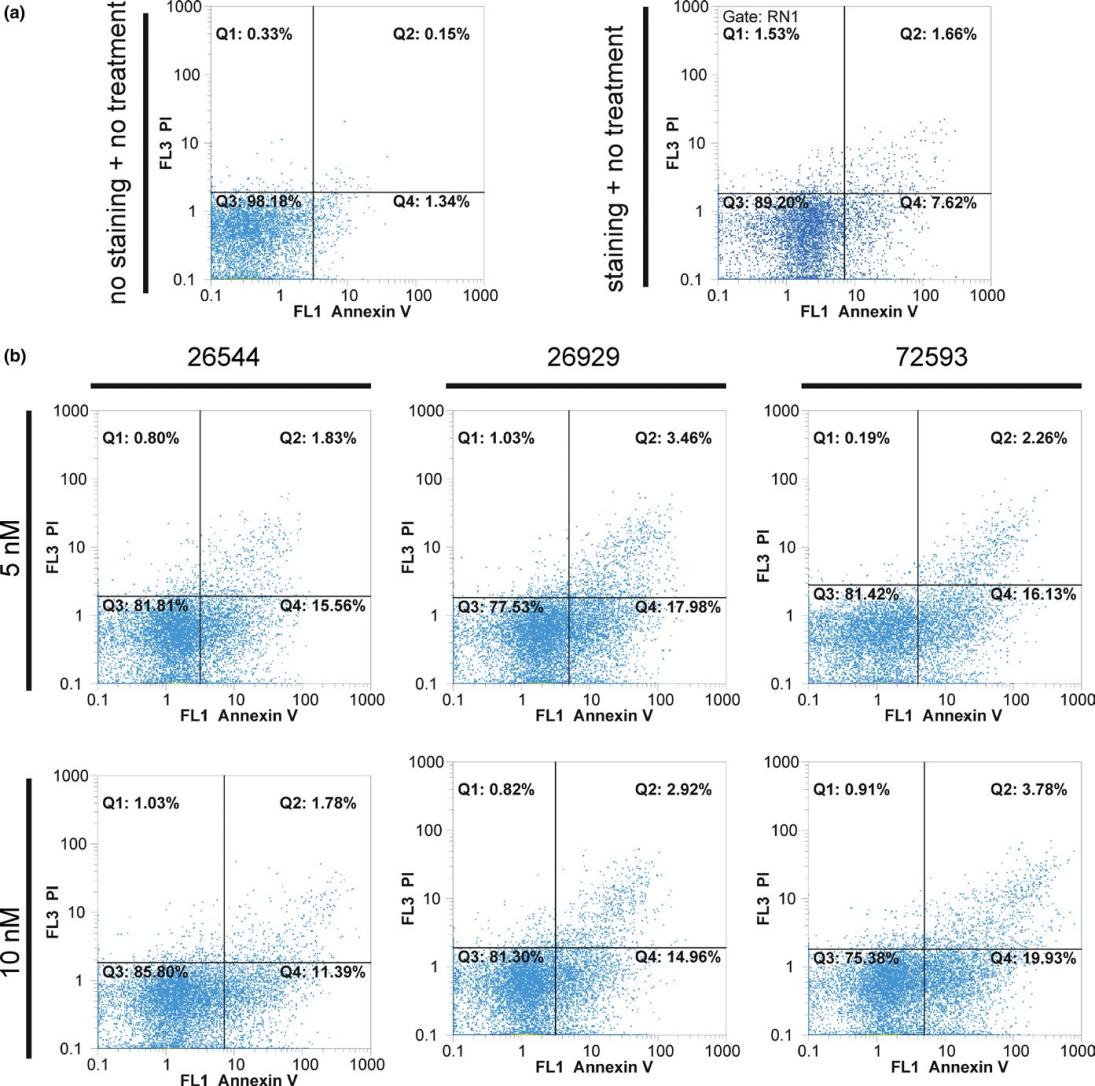
Flow cytometric analysis of apoptosis/necrosis by AnnexinV/Propidium iodide (PI) staining. (a) Staining of controls (not stained and not treated, and stained nontreated). (b) Flow‐cytometric analysis of cell lines treated with 5 and 10 nmol/L of tested agents. Note the differences in AnnexinV+/PI+ (Q2) and Annexin V+/PI‐ (Q4) populations

The AnnexinV+/PI+ (Q2 quadrant) depicts late necrosis and dying cells. The rate of the necrosis triggered by tested compounds did not exceed 4% (only showing a 2.12% increase, compared to the stained control). The Annexin V+/PI‐ (Q4) population represents apoptotic cells or early oncotic cells (see Figure 2). The largest amount of apoptotic or early oncotic cells was found in 10 nmol/L STOCK1N‐72593 after treatment (19.93%; 12.31% increase compared to the stained control). Nevertheless, some portion of oncotic cells is able to reverse oncosis and could thereby survive; thus, no severe increase in the total number of dying cells was found after treatment with tested compounds (see Figure 2).

**Figure 3:**
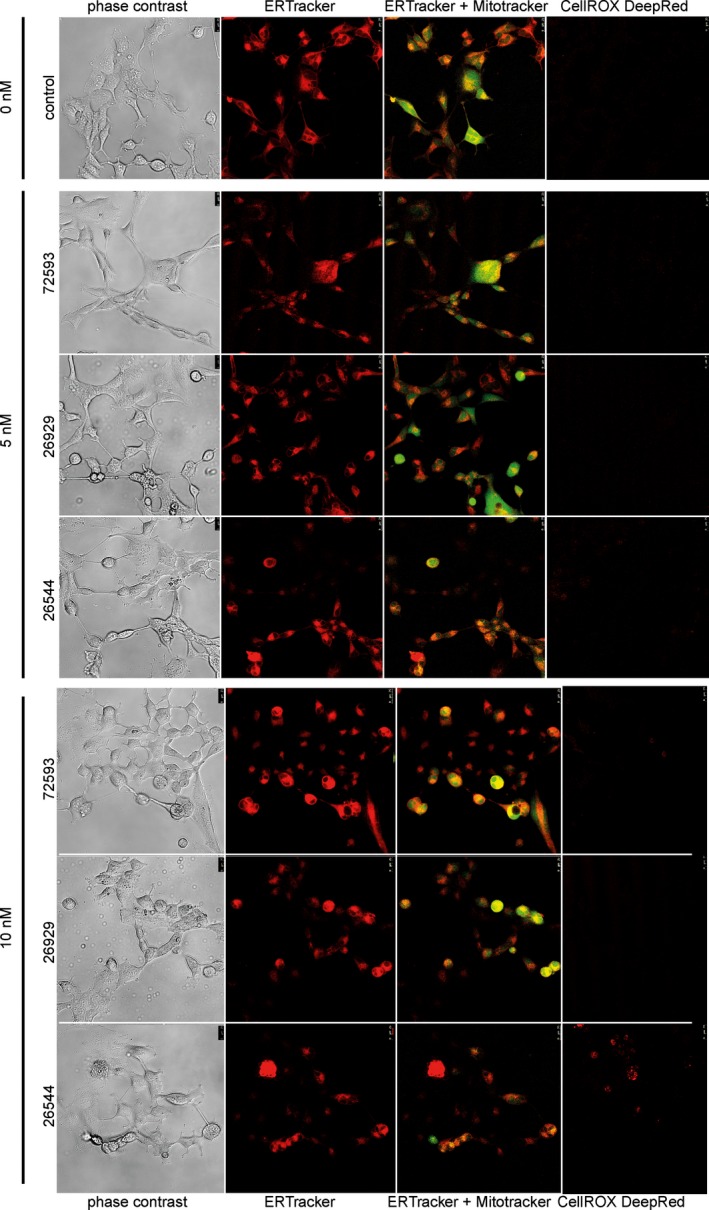
(Fluorescence microscopy analysis of mitochondria and endoplasmic reticulum (ER). ER is stained with red ER tracker, mitochondria) [MR1] with green MitoTracker. Reactive oxygen species were visualized using CellROX Deep Red reagent

Confocal microscopy revealed no significant morphological changes that had been induced by studied compounds. On the other hand, we used two probes to localize mitochondria and the endoplasmic reticulum. Whereas MitoTracker Green FM localizes mitochondria regardless of mitochondrial membrane potential, ER‐Tracker Red, a conjugate of green‐fluorescent BODIPY^®^ TR dye and glibenclamide, also reflects expression of sulphonylurea receptors of ATP‐sensitive K^+^ channels and thereby reflects the ER function. The double‐staining revealed no significant decrease in fluorescence corresponded to both mitochondria and ER for the tested compounds. Compound 26544 showed some ability to generate ROS, which were colocalized with mitochondria (see Figure 3). Nevertheless, ROS production was rather weak.

### 3.2 Benzothiazolone‐2 enhances expression of MT‐3 in microglia cells

**Figure 4:**
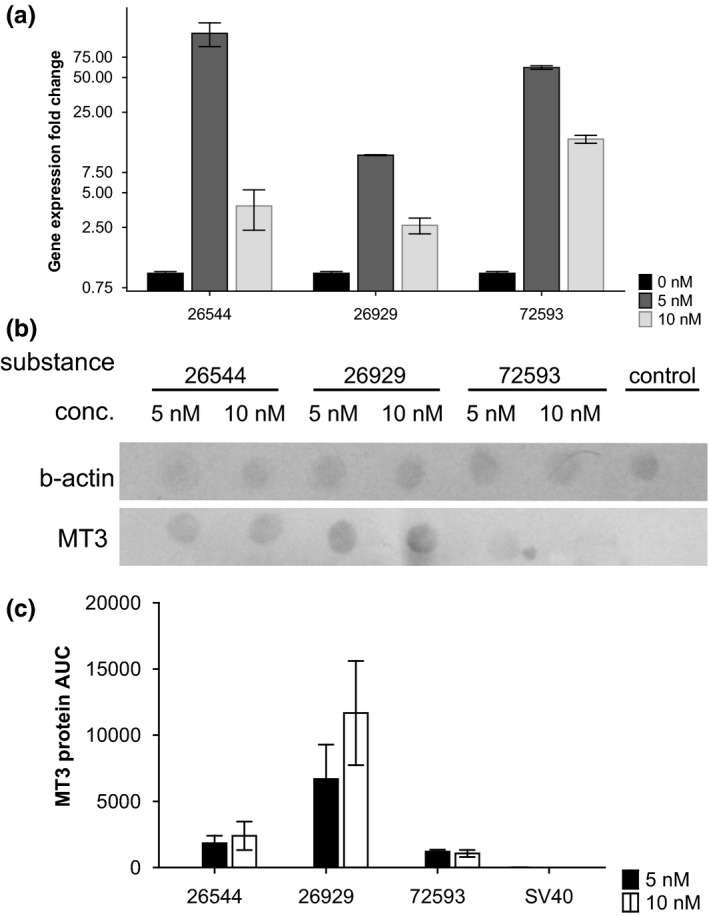
Expression analysis of MT‐3 gene. (a) mRNA expression fold change in cell lines treated with 0, 5, and 10 nmol/L of selected compounds. (b) Dot blot for MT‐3. (c) (Protein level of MT‐3 based on dot blot detection; displayed as mean and standard deviation)

mRNA expression of MT‐3 was relatively low in control cells without treatment. All the three tested compounds enhanced expression of MT‐3 mRNA in both tested concentrations (5 nmol/L and 10 nmol/L, respectively) (see Figure 4a).

Expression of MT‐3 protein was undetectable in control cells without treatment. All the three tested compounds enhanced expression of MT‐3 protein (see Figure 4b). Nevertheless, compound 72593 caused visible enhancement only in 5 nmol/L concentration. Other compounds enhanced expression of MT‐3 protein in tested concentrations (see Figure 4c).

### 3.3 Thermodynamic studies on MT‐3‐benzothiazolone complexes

Binding free‐energy studies that validate the above findings were also performed. The analysis was carried out for the Benzothiazolone‐2 docked complexes only, since the above results were encouraging. The molecular docking representation is provided in Figure S1 It clearly states that the tested molecules (STOCK1N‐26929 and STOCK1N‐26544) both have stable binding free‐energies values in the form of potential, polar, and nonpolar solvation energies that are comparable to the standard controls (Table 1). It also states that both hydrophobic and electrostatic interactions are important for protein ligand interaction in case of MT‐3 for Alzheimer's disease.

**Table 1:** Binding free‐energy calculation of the tested compounds and the standard controls

Ligand	Van der Waal energy (kJ/mol)	Electrostatic energy (kJ/mol)	Polar solvation energy (kJ/mol)	SASA energy (kJ/mol)	Binding energy (kJ/mol)
BDBM50342769	−129.891 ± 5.824	−25.876 ± 5.056	71.608 ± 5.845	−11.920 ± 0.763	−96.078 ± 11.107
BDBM9019	−83.033 ± 9.812	−13.835 ± 4.606	46.691 ± 13.885	−8.855 ± 1.547	−59.033 ± 11.597
BDBM50260394	−101.236 ± 10.190	−42.368 ± 20.609	98.840 ± 35.764	10.603 ± 0.848	−55.367 ± 20.694
STOCK1N‐26544	−60.097 ± 41.578	−92.875 ± 62.292	97.052 ± 69.708	−7.644 ± 5.449	−63.564 ± 40.740
STOCK1N‐ 26929	−107.860 ± 7.382	−71.870 ± 14.138	118.127 ± 28.044	−9.691 ± 0.886	−71.294 ± 21.520

## 4 DISCUSSION

The possibility to enhance the expression of MT‐3 or protect it from degradation is an attractive therapeutic target, because low levels of MT‐3 were found in AD brains. In this study, we tested an enhancement of MT‐3 cellular concentration after MT‐3 binding treatment, which could prevent MT‐3 degradation.

The three compounds under investigation were STOCK1N‐26929, STOCK1N‐26544 (Benzothiazolone‐2 as fragment), and STOCK1N‐72593 (modified tryptamine‐based).

The expression of MT‐3 protein was undetectable in control cells without treatment. All three tested compounds enhanced concentration of MT‐3 protein in cells and surprisingly also mRNA concentration. Nevertheless, STOCK1N‐72593 was relatively less effective**.** It is to be noted here that different concentrations of tested compounds can influence expression of MT‐3 differently. Epigenetic mechanisms and different types of activation of signaling pathways are suspected in this case. Furthermore, the expression of MT‐3 could be regulated at the posttranscriptional level, which was also observed in earlier studies (Garrett et al., 2005). The experiments carried out did not assume that triggering of MT‐3 expression should be in positive correlation with tested compound concentration. The results showed that our compounds are able to influence MT‐3 expression. It is to be mention that our study is not oriented toward exact mechanism or to show exact quantities of MT‐3 protein. In our opinion, two of our compounds significantly enhanced MT‐3 protein and mRNA levels.

The three tested molecules (STOCK1N‐26544, STOCK1N‐26929, and STOCK1N‐72593) have no severe cytotoxic effects on neural tissue (Figure 4). IC50 values of tested molecules exceeded about 10 times the concentration that was needed for induction of MT‐3 expression (Figure 1). Our goal of the precise experiment is the assessment of IC50 value. The findings also demonstrate that concentrations of tested compounds, which are able to trigger MT‐3 synthesis, are not toxic. The experiments carried are not focused toward inhibition of microglia. Our compounds enhanced apoptosis and necrosis, but it was not of severe effect. About 80% of cells were still viable. The rate of the necrosis triggered by tested compounds did not exceed 4% (only showing a 2.12% increase, compared to the stained control). The largest amount of apoptotic or early oncotic cells was found in 10 nmol/L STOCK1N‐72593 after treatment (19.93%; 12.31% increase compared to the stained control).

Flow cytometric analysis on apoptosis/necrosis showed that a lower dose (5 nmol/L) induced a higher toxic effect when compared with higher doses 10 nmol/L. This increase was not the severe one (only about 4%). Epigenetic mechanisms and different types of activation of signaling pathways are suspected in this case. As was shown in other studies, mechanisms of drug‐induced cell death are often concentration dependent. Crosstalk between apoptosis, necrosis, and autophagy could be different in different drug concentrations and numbers of apoptotic cells could be attenuated because of some protective autophagy effects (Kemp, 2017; Miyoshi et al., 2008; Teng et al., 2016; Torres & Horwitz, 1998).

According to fluorescence staining, no serious ROS generation and no severe decrease in mitochondria numbers or endoplasmic reticulum changes after test‐treatment were found. This is very important, (because oxidative stress, mitochondrial dysfunction, and endoplasmic reticulum stress have been implicated in beta‐amyloid neurotoxicity).

The above findings were further validated by calculating the binding free‐energy of the docked complexes through molecular dynamics studies. The MM‐PBSA method uses three energetic terms in order to calculate changes in the free‐energy on binding. The g_mmpbsa tool developed by Kumari et al. (2014), Baker et al. (2001) (http://rashmikumari.github.io/g_mmpbsa/) is used to calculate the MM‐PBSA from each complexes combine with GROMACS. The experiment was run for 1000 ps; several thermodynamic parameters (including RMSD, RMSF, SASA, and H‐bond) have already been reported (Roy et al., 2015). g_mmpbs fetches information from the GROMACS trajectory file and calculates the total free‐energy of the protein‐ligand complex, as well as the free‐energy of protein and ligand, individually. It also calculates the potential energy of molecular mechanics and free‐energy of solvation except entropy contribution.

Interaction energy for three control molecules with STOCK1N compounds was calculated with the MT‐3 receptor, in order to compare it with the active binder. BDBM50342769, BDBM9019, BDBM50260394 binding energies were approximately −96, −59, and −55 kJ/mol. If we look into the individual component of predicted free‐energy, van der Waals energies were dominant in the BDBM50342769, BDBM50260394 binding with MT‐3. Similarly STOCK1N‐26929 showed higher van der Waals energy as well as good binding free‐energy compared to STOCK1N‐26544 (low contribution of van der Waals energy). This suggests one of the major MT‐3 binding interactions is governed by the hydrophobic forces. The second major component was electrostatic energy, which is comparably higher in STOCK1N molecules than in controls.

The overall findings suggest that hydrophobic and electrostatic interaction is imperative for current MT‐3 ligand interaction. Polar solvation and nonpolar solvation energies are comparable to the standard controls (Table 1).

## 5 CONCLUSIONS

The current work has made an effort to see the effect of Benzothiazolone‐2 on the cellular concentration of MT‐3. Many studies show that MT‐3 mRNA is downregulated in AD brains and that this might, therefore, contribute to the upsurge of abnormal neuronal development associated with the disease.

Our previous findings suggests that Benzothiazolone‐2 possesses better pharmacokinetic and pharmacodynamics properties. It also forms stable complex with MT‐3 according to molecular dynamics findings. The above findings were validated by screening the compounds against live immortalized human microglia cells (SV40). This tested compound showed neither severed cytotoxicity nor did it trigger necrosis. In addition, there was no serious ROS‐generation, decrease in mitochondrial numbers, stress‐induced endoplasmic reticulum changes after test‐treatments**.** All three tested compounds enhanced cellular concentration of MT‐3 protein in cells and surprisingly also mRNA expression.

## COMPETING FINANCIAL INTERESTS

We certify that we have no affiliation with or financial involvement with any organization or entity with a direct financial or any other interest in the subject matter or materials discussed in the manuscript.
