# Supplementary figures and images for: The effect of Benzothiazolone‐2 on the expression of Metallothionein‐3 in modulating Alzheimer's disease
Source: Brain Behav. 2017 Aug 15;7(9):e00799. doi: 10.1002/brb3.799 (PMC5607561; doi:10.1002/brb3.799)

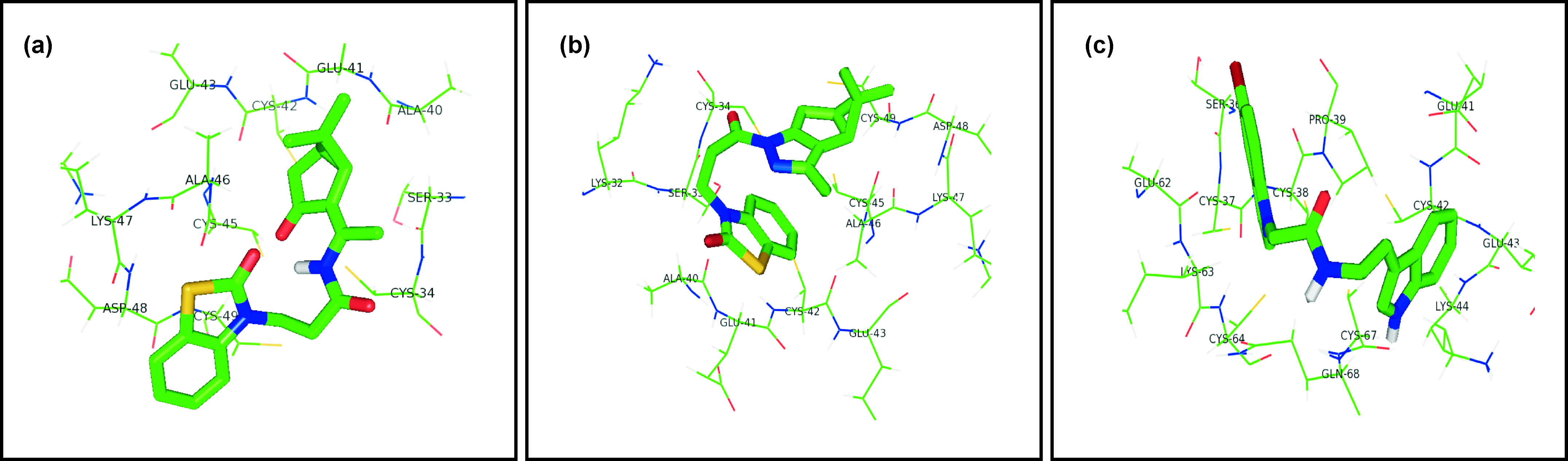

Supplement: Supplementary file 1 [file BRB3-7-e00799-s001.tif]
